# Design, synthesis, and evaluation of *Bothrops* venom serine protease peptidic inhibitors

**DOI:** 10.1590/1678-9199-JVATITD-2020-0066

**Published:** 2021-01-15

**Authors:** Gloria Maria da Silva, Daniel Henrique Berto de Souza, Karoline B. Waitman, Matteo Celano Ebram, Melissa R. Fessel, Iuliu Cezar Zainescu, Fernanda C. Portaro, Montse Heras, Sonia A. de Andrade

**Affiliations:** 1 Laboratory of Pain and Signaling, Butantan Institute, São Paulo, SP, Brazil.; 2 Laboratory of Molecular Biology, Butantan Institute, São Paulo, SP, Brazil.; 3 Department of Chemistry, University of Oxford, Oxford, United Kingdom.; 4 Laboratory of Immunochemistry, Butantan Institute, São Paulo, SP, Brazil.; 5 Laboratory of Innovation in Processes and Products of Organic Synthesis, Department of Chemistry, University of Girona, Montilivi Campus, Girona, Spain.

**Keywords:** Peptides, Snake venom, Serine protease, Disease, Hemostasis

## Abstract

**Background:**

In Central and South America, snakebite envenomation is mainly caused by *Bothrops* spp. snakes, whose venoms feature significant biochemical richness, including serine proteases. The available bothropic antivenoms are efficient in avoiding fatalities, but do not completely neutralize venom serine proteases, which are co-responsible for some disorders observed during envenomation.

**Methods:**

In order to search for tools to improve the antivenom’s, 6-mer peptides were designed based on a specific substrate for *Bothrops jararaca* venom serine proteases, and then synthesized, with the intention to selectively inhibit these enzymes.

**Results:**

Using batroxobin as a snake venom serine protease model, two structurally similar inhibitor peptides were identified. When tested on *B. jararaca* venom, one of the new inhibitors displayed a good potential to inhibit the activity of the venom serine proteases. These inhibitors do not affect human serine proteases as human factor Xa and thrombin, due to their selectivity.

**Conclusion:**

Our study identified two small peptides able to inhibit bothropic serine proteases, but not human ones, can be used as tools to enhance knowledge of the venom composition and function. Moreover, one promising peptide (pepC) was identified that can be explored in the search for improving *Bothrops* spp. envenomation treatment.

## Background

Nearly 3 million of snake envenomation cases, resulting in around 100 thousand deaths, and about 400 thousand amputations and other sequelae, are estimated to occur *per* year in the world [1], constituting a serious public health issue. Most snakebite envenomation cases in Central and South America are caused by snakes from the *Bothrops* genus. Typical clinical symptoms of envenomation caused by *Bothrops* spp*.* snakebites include pain, blisters and hemostatic disturbances, and in more severe cases, abscesses, necrosis, and acute kidney failure [2]. These manifestations are mainly associated with the action of metalloproteases (SVMPs) and serine proteases (SVSPs) [3]. 

SVSPs are the second most abundant enzymes from snake venom protein content, comprising in average 20.6% of it, reaching up to 36% in some individuals [4,5]. SVSPs have been reported to affect platelet aggregation [6] and human blood coagulation [7]. Some SVSPs display fibrinogenolytic activity and are classified as thrombin-like enzymes (TLEs) [8]. The catalytic triad is highly conserved among TLEs and human serine proteases (hSPs), such as human thrombin. However, TLEs usually do not cleave both chains of fibrinogen, neither activate other coagulation factors such as factor XIII, causing an imbalance in the victim's hemostatic system [8]. 

The administration of specific antivenom is the standard treatment for snakebite, as recommended by the World Health Organization [1]. Bothropic antivenom is usually produced by horse immunization with a mixture of venoms from different *Bothrops* species. Recent studies indicate that antivenoms used in the treatment of snakebites are very efficient in neutralizing the actions of SVMPs, but do not completely neutralize SVSPs [9]. It was demonstrated that enzymatic activities from proteins bigger than 50kDa, such as some SVMPs, were efficiently neutralized by polyvalent antivenom (PAV); however, activities of enzymes smaller than 50kDa, such as SVSPs, were poorly neutralized by PAV. Interestingly, most of these proteins were well recognized by PAV, suggesting that the catalytic sites of the enzymes may be poor immunogens [10]. 

In this context, the use of selective SVSPs inhibitory compounds, in association with available commercial antivenoms, could be a starting point for studies aiming snakebite treatment efficacy improvement. Therefore, in the present study, small peptides were evaluated as potential SVSPs inhibitors. Based on a previously described substrate, Abz-Ser, selective for *Bothrops* spp venom SVSPs and able to detect PAV non-neutralized serine protease activities on *Bothrops jararaca* venom (BjV) [9], 6-mer peptides were designed, synthesized, characterized, and tested on batroxobin, a commercial protein used as a SVSP model, and on BjV. The goal of this work is the identification of *Bothrops* SVSPs inhibitory peptides, inert to hSPs, which can be used as tools for increasing bothropic SVSPs knowledge and, in perspective, in combination to antivenom for treatment improvement and enhancement of the envenomed patient’s clinical picture.

## Methods

### Materials

The amino acids and the PEG resin were provided by Novabiochem^®^. Hydroxybenzotriazole (HOBt), piperidine, formic acid, O-Benzotriazol-1-yl)-N,N,N,N-tetramethyluronium hexaﬂuorophosphate (HBTU), N-methyl- morpholine (NMM), 1,2-ethanedithiol (EDT), phenol, thioanisol, N,N′-Diisopropylcarbodiimide (DIC), Triisopropylsilane (TIS), Trifluoroacetic acid (TFA), ether, human trypsin and trypsin substrate Nα-Benzoyl-DL-arginine 4-nitroanilide hydrochloride (BAPNA) were provided by Sigma Aldrich^®^. VWR Chemicals^®^ provided the Dimethylformamide (DMF) and acetonitrile (ACN). Fmoc-Rink MBHA resin and reagent Oxyma Pure were provided by Iris Biotech. Human factor Xa, thrombin, and their chromogenic substrates, Bz-Ile-Glu (γ-OCH_3_)-Gly-Arg-pNA [CS-11(22)] and H-D-Phe-Pip-Arg-pNa, 2HCl [CS-01(38)], respectively, were provided by Hyphen. Batroxobin was provided by Pentapharm. *Bothrops jararaca* venom (BjV) was supplied by the Herpetology Laboratory from Butantan Institute (IB), São Paulo, Brazil. The fluorogenic substrate Abz-Ser (Abz-RPPGFSPFRQ-EDDnp) was provided by Dr. Fernanda C.V. Portaro.

### Peptide synthesis, purification and identification by mass spectrometry

Three 6-mer peptides, named pepA, pepB, and pepC, were designed based on the C-terminal of Abz-Ser, a synthetic peptide based on human kininogen sequence, previously reported as substrate for *Bothrops* spp venom-specific serine protease-like enzymes [9]. Peptides pepA and pepC were synthesized using automated solid-phase multiple synthesizer (Peptide Synthesizer PSSM 8-Shimadzu) with the Fmoc strategy [11]. The synthesis of peptide pepB was carried out manually using the solid-phase technique in polypropylene syringes equipped with a polyethylene filter using standard Fmoc/t-Butyl chemistry. A Fmoc-Rink-MBHA resin (181 mg, 0.71 mmol/g) was treated with CH_2_Cl_2_ (1 x 20 min) and with DMF (1 x 20 min). Fmoc group removal was achieved with piperidine-DMF (3:7, 2 + 10 min). Coupling of commercial Fmoc-amino acids (4 equiv) were performed using DIC (4 equiv) and Oxyma Pure (4 equiv) in DMF under stirring at room temperature for 2 h and monitoring by Kaiser test. For each coupling and deprotection step, the resin was washed with DMF (6 x 1 min) and CH_2_Cl_2_ (3 x 1 min), and air-dried. Peptide elongation was performed by repeated cycles of Fmoc removal, coupling and washings. Once the synthesis was completed, peptidyl resin was subjected to the N-terminal Fmoc removal. 

All peptides were cleaved by treatment with TFA-H_2_O-TIS (95:2.5:2.5) for 2h. Following, TFA was evaporated by bubbling N_2_ into the solution. Crude peptides were precipitated twice by adding cold diethyl ether (-20ºC) and collected by centrifugation. Next, the peptide was dissolved in H_2_O-CH_3_CN (1:1 v/v containing 0.1% TFA), and lyophilized. The peptides were purified in HPLC system (Shimadzu, Co., Japan) using a reverse phase column (Shim-pack Prep-ODS, 15 µm x 20 x 250 mm) (available at Additional file 1).

### Peptide analysis by mass spectrometry

Synthesized peptides were analyzed by mass spectrometry, through direct injection on positive module (ESI^+^) using a Finnigan Surveyor MSQ Plus (Thermo Fisher Scientific Inc., Waltham, MA, USA) mass spectrometer with the following analysis parameters: solvent A (H_2_O/formic acid 1000:1); solvent B (ACN/H_2_O/formic acid - 800:200:1); solvent C (Methanol/ H_2_O 800:200); cone voltage: 40V; temperature 150°C and nitrogen at 75 psi as it was identified gas.

### Peptide characterization by NMR

All peptides were characterized by ^1^H-NMR and COSY spectra. The experiments were recorded on a Bruker Ultrashield Avance 400 MHz instrument. Chemical shifts were reported as δ values (ppm) directly referenced to the solvent signal. Coupling constants (*J*) are given in Hertz (Hz). The following abbreviations were used for spin multiplicity: s=singlet, d=doublet, t=triplet, q=quartet, m=multiplet, dd=doublet of doublets. 

### Enzymatic assays

Batroxobin and human thrombin activities were evaluated using the chromogenic substrate S-2238 (Chromogenix). Briefly, batroxobin (0.8 U) or human thrombin (0.8U) was pre-incubated at 37°C for 10 minutes with different concentrations (0 to 1 mg/mL for batroxobin, and 60 µg/mL, for human thrombin), of each peptide in Tris/HCl buffer 0.15mM, containing CaCl_2_ 0.02% (v/v), pH 8.0. Enzymatic reaction was initiated by addition of substrate (200µM), followed by incubation at 37°C, for 20 minutes. When serine protease activity from BjV was evaluated using the chromogenic substrate S2238, 5 µg of BjV and 60 µg/mL of each peptide were employed.

Proteolytic activity of human factor Xa was evaluated using the chromogenic substrate CS-11(22) (Hyphen). Briefly, factor Xa (20nM) was pre-incubated for 10 minutes at 37°C with 60 µg/mL of each peptide in Tris/HCl buffer 20 mM, pH 7.4 containing NaCl 150 mM. Enzymatic reaction was initiated by the addition of substrate (100µM), followed by incubation at 37°C, for 20 minutes.

Proteolytic activity of human trypsin was evaluated using the chromogenic substrate BAPNA (Sigma). Briefly, trypsin (126 nM) was pre-incubated for 10 minutes at 37°C with 60 µg/mL of each peptide in Tris/HCl buffer 50 mM containing CaCl_2_ 0.02% (v/v), pH 8.0. Enzymatic reaction was initiated by addition of substrate (1.25 mM), followed by incubation at 37°C, for 20 minutes. 

For all assays using chromogenic substrates, hydrolysis was quantified by evaluation of absorbance at 405 nm, in a spectrophotometer SpectraMax^®^ Paradigm^®^ Multi-Mode reader from Molecular Devices (Sunnyvale, CA).

SVSPs activity from BjV was also evaluated using the fluorogenic substrate Abz-Ser. Briefly, BjV (5 µg/100µL) was pre-incubated at 37^o^C for 10 minutes with 100 µg/mL of each peptide, in PBS pH 7.4 containing 20mM NaCl. Enzymatic reaction was initiated by addition of substrate (10 µM), followed by incubation at 37°C, for 20 minutes. The substrate hydrolysis was monitored following fluorescence emitted at 420 nm, under excitation at 320 nm in a spectrofluorimeter (Victor 3^TM^ Perkin- Elmer, Boston, MA, USA), as described by Araujo et al. (2000) [12]. All the experiments were made in triplicate. 

## Results

### **Design, synthesis, purification, and characterization of *Bothrops* serine protease inhibitory peptide candidates**

**Figure 1.**
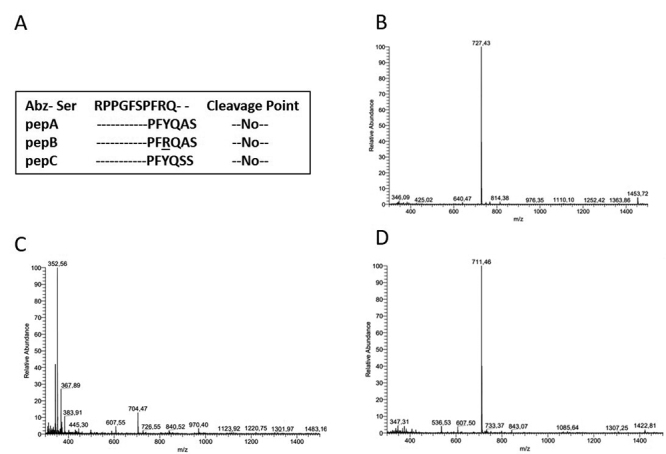
Design and mass spectrum analyses of venom serine protease inhibitor candidates. **(A)** Multialignment of specific venom serine protease substrate (Abz-Ser) [9] with derived peptides (pepA, pepB, pepC). **R** on pepB indicates D-Arg. The primary sequences of these peptides were subjected to *in silico* hydrolysis with the human serine proteases (thrombin, trypsin and factor Xa) using ExPASy PeptideCutter program [2]. No cleavage points were observed in these conditions. **(B, C, D)** Mass spectrum of purified peptides. Insets on (B) to (D): corresponding peptide structures.

Based on carboxyl-terminus of the substrate Abz-Ser, a 6-mer peptide was designed, named pepA (Pro-Phe-Tyr-Gln-Ala-Ser). Then, related to pepA, another two peptides were designed, each one containing one-amino acid substitution: pepB presents a D-Arg replacing Tyr (Pro-Phe-D-Arg-Gln-Ala-Ser), and pepC, a Ser instead of Ala (Pro-Phe-Tyr-Gln-Ser-Ser). Figure 1 shows the alignment of the peptidic portion of the substrate Abz-Ser with the derived peptides.

Peptides pepA, pepB, and pepC were synthesized, purified, and submitted to mass spectrometry analyses (Figure 1B to 1D). PepA mass spectrum revealed a single peak at 711.46 m/z, compatible with its predicted molecular weight (710.70 Da) and charge +1. Mass spectrum of pepB presented a major peak at 352.56 m/z, consistent with its predicted molecular weight (703.80 Da) and charge +2. For pepC, mass spectrum showed a single peak at 727.43 m/z, in agreement with its predicted molecular weight (727.32 Da) and charge +1. 

The ^1^H-NMR spectra of the purified peptides were performed. PepA and pepC spectra present, in the aromatic region, the characteristic signals (two doublets) of the phenol group from tyrosine side chain. Accordingly, these signals are not present in the pepB spectrum. However, the signal of the δ-methylene group of the arginine side chain can be distinguished around 3.4 ppm as a multiplet. On the other hand, pepA and pepB spectra present a doublet signal around 1.5 ppm corresponding to methyl group of the alanine side chain. PepC contains a serine at position five instead of an alanine, and in its spectrum the signal of the two β-methylene group of the serine side chains can be appreciated as a multiplet around 4.8 ppm in the pepC spectrum (available as Additional files 2 and 3). With the help of COSY experiments all signals of the ^1^H-NMR spectra were assigned unequivocally. These results confirmed that the three peptides were synthesized with the desired sequence (available at Additional file 4).

### Evaluation of synthetic peptides on batroxobin thrombin-like and on human serine proteases activities

**Figure 2.**
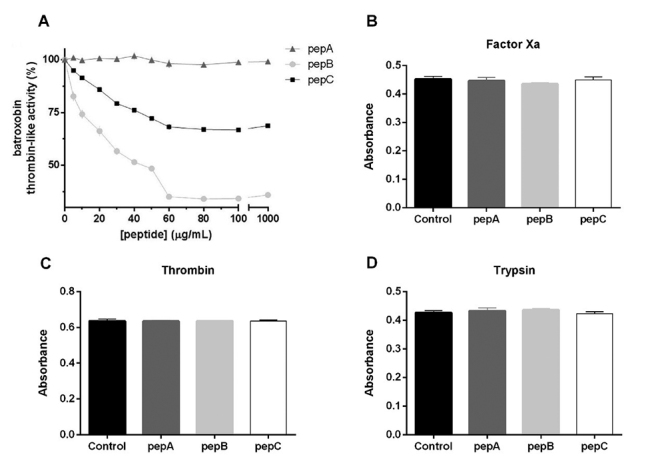
Evaluation of the effects of synthetic peptides on batroxobin and on human serine proteases activities. **(A)** Batroxobin activity was evaluated after 20 min, at 37^o^C, using the chromogenic substrate S-2238 and the indicated concentrations of synthetic peptides. **(B, C, D)** Commercial hSPs had their activity evaluated after 20 min, at 37^o^C, using their specific chromogenic substrates and batroxobin inhibitory peptides at 60 µg/mL.

Thrombin-like activity of batroxobin was evaluated using a chromogenic thrombin substrate (S2238) in the presence of increasing concentration of the peptides. Peptide pepA did not inhibit batroxobin activity when tested until 1 mg/mL (Figure 2A). PepB, which features a single amino acid change regarding pepA (D-Arg replacing a Tyr residue) was effective on batroxobin inhibition with 60 µg/mL (36 ± 7%; Figure 2A). Peptide pepC, which contains another single modification related to pepA (a Ser replacing an Ala residue) was also able to inhibit batroxobin with a concentration of 60 µg/mL (18 ± 4%; Figure 2A). 

Facing the catalytic triad similarity among serine proteases, and the importance of not inhibiting the human counterparts to achieve balanced hemostasis, the human factor Xa, thrombin, and trypsin had their serine protease activities evaluated. When 60 µg/mL batroxobin inhibitory peptide was used, no significant effect was observed on the catalytic activities of human ortholog serine proteases (Figure 2B). 

Additionally, the primary sequences of these peptides were subjected to *in silico* hydrolysis with the human serine proteases (thrombin, trypsin and factor Xa (Figure 1A) using ExPASy PeptideCutter program [24]. No cleavage points were observed in these conditions.

### **Evaluation of synthetic peptides on *Bothrops* venom serine proteases**

**Figure 3.**
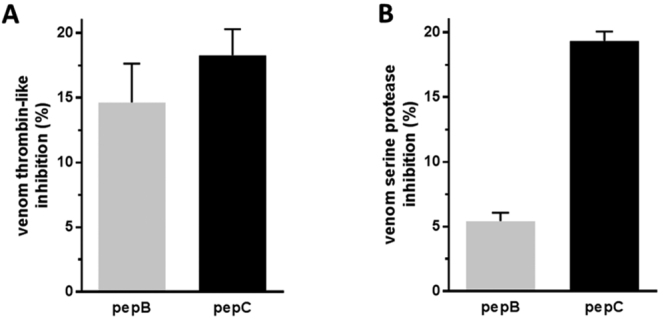
Evaluation of synthetic peptides on *Bothrops* venom serine-proteases. BjV was pre-incubated with 100 µg/mL of pepB or pepC and a serine proteases activity was evaluated after 20 min, at 37^o^C, with **(A)** 2 mM of substrate S2238 or **(B)** 10 µM of substrate Abz-Ser.

Inhibitory potential of peptides pepB and pepC was analyzed using BjV as source of serine proteases. Using the thrombin chromogenic substrate S2238, it was observed that both peptides were similarly able to inhibit BjV thrombin-like activity (Figure 3A: inhibition of 15 ± 3% and 18 ± 2% for pepB and pepC, respectively). However, when synthetic peptides were tested on BjV using the substrate Abz-Ser, designed based on kininogen sequence and previously described as being selective for *Bothrops* spp. venom serine proteases, (Figure 3B), the inhibitory effect of pepB was 4 times less efficient than pepC (inhibition of 5.4 ± 0.7% and 19.3 ± 0.7% for pepB and pepC, respectively). 

## Discussion

Considering recent studies reporting residual SVSPs activity after bothropic antivenom treatment [9, 10, 13], we decided to search for peptides able to inhibit these proteins, which are enzymes co-responsible for hemostatic disorders observed on snakebites envenomation. The use of peptides as therapeutic molecules is well established, since they can be designed target-specifically to present high specificity and affinity and, additionally, peptides and their metabolites usually present low toxicity levels [14,15]. One strategy for designing protein inhibitors is to use as a base the sequence of natural inhibitors or substrates [16]. In this context, in order to obtain peptides with potential to be SVSP selective inhibitors, we focused on the amino acid sequence of the previously reported *Bothrops* spp venom serine proteases-selective substrate, Abz-Ser, which was also able to detect relevant non neutralized serine protease activity on BjV PAV-treated [9].

Indeed, based on Abz-Ser, three 6-mer peptides were planned according some rational strategies. For peptide pepA, conserved residues related to kininogen were maintained. For peptide pepB, it was taken into consideration the proteolytic susceptibility of peptides containing only L-amino acids and the fact that D-amino acids-containing peptides rarely act as substrates of endogenous proteases [21], and a single change regarding pepA was designed: D-Arg instead of a Tyr residue. Finally, since SVSPs usually present a hydrophobic region outside the catalytic site [8] which could favor the selective inhibition of venom enzymes avoiding the highly conserved catalytic triad [7,22,23], peptide pepC was designed containing another substitution regarding to pepA - a Ser replacing an Ala residue - in order to evaluate if an alteration on the peptide hydrophobicity could affect its effects on the enzyme.

These 6-mer peptide were synthetized, purified, and analyzed by mass spectrometry and ^1^H-NMR. In addition to confirm peptides identities, both analytical techniques revealed that purified peptides were obtained with high level of purity. No other diastereoisomer could be detected by ^1^H-NMR, which indicates that epimerization processes did not took place during the peptide synthesis. It is easy to appreciate the expected differences in the peptide sequences of pepA, pepB and pepC with a simple analysis of the main signals of their ^1^H-NMR spectra (available as Additional file 2). 

Since bothropic SVSPs activities are mainly related to TLEs [7,17], a commercially available TLE from *B. moojeni* snake venom [18,19], 90% identical to bothrombin, the *B. jararaca* venom ortholog [20], the enzyme batroxobin, was used as a model protein to evaluate the effects of the synthetic peptides on serine protease activity.

Interestingly, while pepA was not able to affect batroxobin thrombin-like activity, both pepB and pepC inhibited its SP activity, being pepB twice more efficient than pepC. It is worthy to note that, although being less hydrophobic than the non-inhibitory peptide pepA, pepC was able to inhibit batroxobin. Importantly, none of the peptides inhibited human serine proteases indicating specificity of the inhibitory activities of pepB and pepC.

Following, peptides pepB and pepC were evaluated as potential BjV SVSPs inhibitors using the thrombin S2238 substrate and, indeed, they were able to inhibit thrombin-related SVSP activity similarly. Curiously, when synthetic peptides were tested on BjV using Abz-Ser, inhibitory effect of pepB was 4 times less efficient than pepC. 

The differences between the inhibition levels obtained when batroxobin was used, in comparison with the results obtained with BjV, possibly demonstrate the molecular complexity of the *B. jararaca* venom [5], suggesting the contribution of different SVSPs activities in the venom. Taken together, these data indicate that pepB is a good inhibitor for bothropic TLEs, as batroxobin, and, suggest that, on SVSP activity evaluated with Abz-Ser, this enzyme category has a low contribution to the non-neutralized serine protease pool. On the other hand, pepC was able to inhibit BjV TLE activity as well the BjV SVSPs tested with Abz-Ser. Further assays are necessary to determine the identity of these proteins and pepC can be a good tool to achieve it.

Additionally, the substrate Abz-Ser demonstrated that commercial PAV was unable to neutralize nearly 70% of BjV SVSPs [9]. Therefore, Abz-Ser can be considered a good substrate to search for candidates capable of inhibiting its hydrolysis, since it is little blocked by the commercial PAV.

Since antibody recognition can be insufficient to inhibit this enzyme class, assays as ELISA of venom PAV non-neutralized fraction could still display active antibody-bounded enzymes relevant to the clinical picture of snakebite envenomation. Thus, PepC can be a tool for search for SVSPs not inhibited by PAV, in a simple way, and to elucidate the identity of these proteins. Meanwhile, pepC, an easy and low cost obtained peptide, could be use as clinical support together with regular immunoterapy so as to improve treatment efficacy.

## Conclusion

In conclusion, our study identified two small peptides able to inhibit bothropic serine proteases, but not human ones, which can be used as tools to enhance knowledge of the bothropic venom composition and function and, importantly, one promising peptide (pepC) to be explored in a search for improving *Bothrops* spp envenomation treatment.

### Abbreviations

BjV: *Bothrops jararaca* venom; hSP: human serine protease; SVMP: snake venom metalloprotease; SVSP: snake venom serine protease; TLE: thrombin-like enzyme. 

## Supplementary material

The following online material is available for this article:

Additional file 1. Chromatography profile of pepA, pepB, and pepC.

Additional file 2.
^1^H-RMN spectra (region 7.5-1.0 ppm) of pepA, pepB, and pepC recorded in CD_3_OD at 400 MHz.

Additional file 3. Copies of NMR spectra. ^1^H-RMN and COSY experiments.

Additional file 4. Copies of NMR spectra (^1^H-RMN).
