# Supplementary material for: Design, synthesis, and evaluation of Bothrops venom serine protease peptidic inhibitors
Source: J Venom Anim Toxins Incl Trop Dis. 2021 Jan 15;27:e20200066. doi: 10.1590/1678-9199-JVATITD-2020-0066 (PMC7810238; doi:10.1590/1678-9199-JVATITD-2020-0066)
Supplement: Additional file 1. [file 1678-9199-jvatitd-27-e20200066-s1.pdf]

J Venom Anim Toxins incl Trop Dis
https://doi.org/10.1590/1678-9199-JVATITD-2020-0066

Supplementary Material to "Design, synthesis, and evaluation of *Bothrops* venom serine protease peptidic inhibitors"

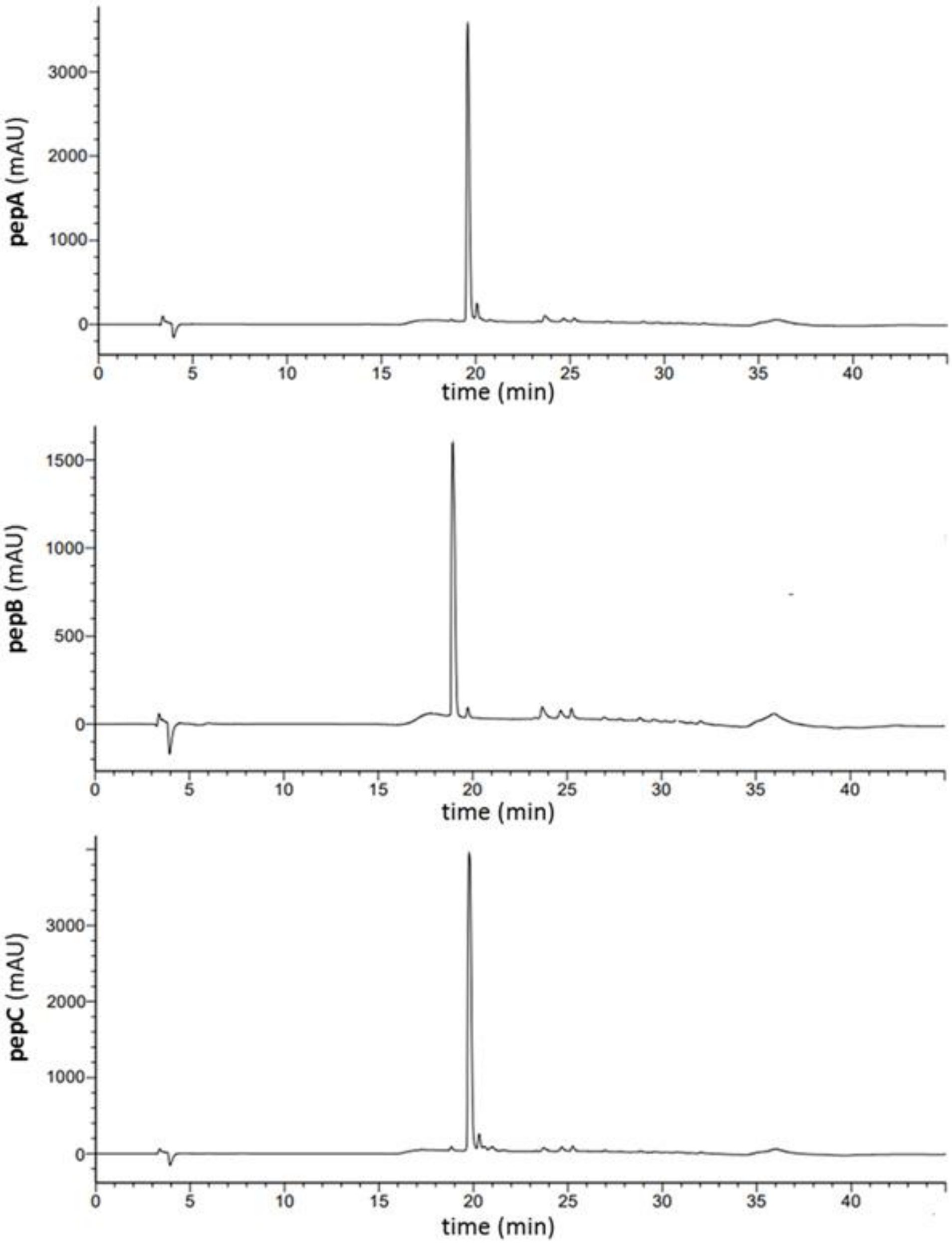

Additional file 1 - Chromatography profile of pepA, pepB, and pepC.
