# Supplementary material for: Design, synthesis, and evaluation of Bothrops venom serine protease peptidic inhibitors
Source: J Venom Anim Toxins Incl Trop Dis. 2021 Jan 15;27:e20200066. doi: 10.1590/1678-9199-JVATITD-2020-0066 (PMC7810238; doi:10.1590/1678-9199-JVATITD-2020-0066)
Supplement: Additional file 4. [file 1678-9199-jvatitd-27-e20200066-s4.pdf]

J Venom Anim Toxins incl Trop Dis
https://doi.org/10.1590/1678-9199-JVATITD-2020-0066

Supplementary Material to "Design, synthesis, and evaluation of *Bothrops* venom serine protease peptidic inhibitors"

Additional file 4 - Copies of NMR spectra (^{1}H-RMN).

^{1}H-NMR of pepA (Pro-Phe-Tyr-Gln-Ala-Ser-NH$_2$):

^{1}H NMR (CD$_3$OD, 400 MHz) δ 7.29-7.19 (m, 5H, H_{arom}-Phe), 7.03 (d, *J*=8.5 Hz, 2H, H_{arom}-Tyr), 6.68 (d, *J*=8.5 Hz, 2H, H_{arom}-Tyr), 4.63 (dd, *J*=9.6, 5.2 Hz, 1H, CH_α-Phe), 4.51 (dd, *J*=8.8, 5.5 Hz, 1H, CH_α-Tyr), 4.39-4.29 (m, 3H, CH_α-Ala, CH_α-Ser and CH_α-Gln), 4.03 (dd, *J*=8.6, 6.8 Hz, 1H, CH_α-Pro), 3.85 (dd, *J*=11.3, 5.4 Hz, 1H, one proton C$H_{2\beta}$-Ser), 3.79 (dd, *J*=11.3, 4.7 Hz, 1H, one proton C$H_{2\beta}$-Ser), 3.27-3.18 (m, 2H, C$H_{2\delta}$-Pro), 3.13 (dd, *J*=14.1, 5.2 Hz, 1H, one proton C$H_{2\beta}$-Phe), 3.05 (dd, *J*=14.0, 5.4 Hz, 1H, one proton C$H_{2\beta}$-Tyr), 2.90-2.83 (m, 2H, one proton C$H_{2\beta}$-Phe and one proton C$H_{2\beta}$-Tyr), 2.37-2.21, (m, 3H, C$H_{2\gamma}$-Gln and one proton C$H_{2\beta}$-Pro), 2.13-1.96 (m, 2H, C$H_{2\beta}$-Gln), 1.94-1.86 (m, 2H, C$H_{2\gamma}$-Pro), 1.82-1.74 (m, 1H, one proton C$H_{2\beta}$-Pro), 1.42 (*d*, J=7.0 Hz, 3H, C$H_{3\beta}$-Ala).

^{1}H-NMR of pepB (Pro-Phe-D-Arg-Gln-Ala-Ser-NH$_2$):

^{1}H-NMR (CD$_3$OD + one drop D$_2$O, 400 MHz) δ 7.35-7.24 (m, 5H, H_{arom}-Phe), 4.48 (t_{ap}, *J*=8.4 Hz, 1H, CH_α-Phe), 4.43-4.30 (m, 4H, CH_α-Ala, CH_α-Arg, CH_α-Ser and CH_α-Gln), 4.12 (dd, *J*=10.0, 4.1 Hz, 1H, CH_α-Pro), 3.86 (dd, *J*=11.6, 5.6 Hz, 1H, one proton C$H_{2\beta}$-Ser), 3.82 (dd, *J*=11.6, 4.8 Hz, 1H, one proton C$H_{2\beta}$-Ser), 3.44-3.36 (m, 2H, C$H_{2\delta}$-Arg), 3.18 (dd, *J*=13.5, 7.8 Hz, 1H, one proton C$H_{2\beta}$-Phe), 3.09 (dd, *J*=13.5, 8.7 Hz, 1H, one proton C$H_{2\beta}$-Phe), 3.07-2.98 (m, 2H, C$H_{2\delta}$-Pro), 2.42-2.31 (m, 1H, one proton C$H_{2\beta}$-Arg), 2.34 (t, *J*=7.8 Hz, 2H, C$H_{2\gamma}$-Gln), 2.18-2.09 (m, 1H, one proton C$H_{2\beta}$-Gln), 2.08-1.95 (m, 3H, one proton C$H_{2\beta}$-Gln and C$H_{2\gamma}$-Arg), 1.91-1.83 (m, 1H, one proton C$H_{2\beta}$-Arg), 1.79-1.71 (m, 1H, one proton C$H_{2\beta}$-Pro), 1.66-1.56 (m, 1H, one proton C$H_{2\beta}$-Pro), 1.43 (d, *J*=7.2 Hz, 3H, C$H_{3\beta}$-Ala), 1.26-1.11 (m, 2H, C$H_{2\gamma}$-Pro).

https://doi.org/10.1590/1678-9199-JVATITD-2020-0066

^{1}H-NMR of pepC (Pro-Phe-Tyr-Gln-Ser-Ser-NH_2):

^{1}H-NMR (CD_3OD, 400 MHz) δ 7.28-7.21 (m, 5H, H_{arom}-Phe), 7.04 (d, *J*=8.3 Hz, 2H, H_{arom}-Tyr), 6.69 (d, *J*=8.3 Hz, 2H, H_{arom}-Tyr), 4.63 (dd, *J*=9.5, 5.2 Hz, 1H, CH_{α}-Phe), 4.51 (dd, *J*=8.8, 5.4 Hz, 1H, CH_{α}-Tyr), 4.45-4.40 (m, 2H, 2 x CH_{α}-Ser), 4.35 (t_{ap}, *J*=7.2 Hz, 1H, CH_{α}-Gln), 4.05 (t_{ap}, *J*=7.3 Hz, 1H, CH_{α}-Pro), 3.93-3.76 (m, 4H, 2 x C$H_{2\beta}$-Ser), 3.27-3.21 (m, 2H, C$H_{2\delta}$-Pro), 3.13 (dd, *J*=14.1, 5.2 Hz, 1H, one proton C$H_{2\beta}$-Phe), 3.06 (dd, *J*=14.1, 5.0 Hz, 1H, one proton C$H_{2\beta}$-Tyr), 2.90-2.83 (m, 2H, one proton C$H_{2\beta}$-Phe and one proton C$H_{2\beta}$-Tyr), 2.38-2.23 (m, 3H, C$H_{2\gamma}$-Gln and one proton C$H_{2\beta}$-Pro), 2.15-1.98 (m, 2H, C$H_{2\beta}$-Gln), 1.96-1.89 (m, 2H, C$H_{2\gamma}$-Pro), 1.84-1.75 (m, 1H, one proton C$H_{2\beta}$-Pro).
